# Simultaneous Determination of Valsartan and Hydrochlorothiazide in Tablets by RP-HPLC

**DOI:** 10.4103/0250-474X.43006

**Published:** 2008

**Authors:** D. F. Tian, X. L. Tian, T. Tian, Z. Y. Wang, F. K. Mo

**Affiliations:** Department of Pharmacy, School of Pharmacy, Shenyang Pharmaceutical University, 110016 Shenyang, China

**Keywords:** Valsartan, hydrochlorothiazide, RP-HPLC, simultaneous determination, tablets

## Abstract

A simple, reproducible and efficient reverse phase high performance liquid chromatographic method was developed for simultaneous determination of valsartan and hydrochlorothiazide in tablets. A column having 200 × 4.6 mm i.d. in isocratic mode with mobile phase containing methanol:acetonitrile:water:isopropylalcohol (22:18:68:2; adjusted to pH 8.0 using triethylamine; v/v) was used. The flow rate was 1.0 ml/min and effluent was monitored at 270 nm. The retention time (min) and linearity range (μg/ml) for valsartan and hydrochlorothiazide were (3.42, 8.43) and (5-150, 78-234), respectively. The developed method was found to be accurate, precise and selective for simultaneous determination of valsartan and hydrochlorothiazide in tablets.

Valsartan, (S)-N-(1-Oxopentyl)-N-[[2’-(1H-tetrazol-5-yl)[1,1’-biphenyl]-4-yl]methyl]-L-valine, is an orally active specific angiotensin II receptor blocker effective in lowering blood pressure in hypertensive patients^1^. A number of high performance liquid chromatographic (HPLC) methods are available for separation and quantification of valsartan from pharmaceutical dosage forms^2^. Hydrochlorothiazide is a diuretic of the class of benzothiadiazines widely used in antihypertensive pharmaceutical formulations, alone or in combination with other drugs, which decreases active sodium reabsorption and reduces peripheral vascular resistance^3^. It is chemically 6-chloro-3,4-dihydro-2H-1,2,4-benzothiadiazine-7-sulfonamide-1,1-dioxide, and was successfully used as one content in association with other drugs^4^–^9^ in the treatment of hypertension. Simultaneous determination of both drugs is highly desirable as this would allow more efficient generation of clinical data and could be more cost-effective than separate assays. There are very few methods appearing in the literature for the simultaneous determination of valsartan and hydrochlorothiazide in tablets. Since these methods were based on HPLC and UV-derivative spectrophotometry^10^–^11^, the procedure was inconvenient for determination and the run times were rather long. The aim of this study was to develop a simple, precise and accurate reverse-base high performance liquid chromatographic method to estimate valsartan and hydrochlorothiazide in tablets. This method was simple and rapid and provides accurate and precise results, as compared with other methods which have been reported. Criteria employed for assessing the suitability of said solvent system were cost-effectiveness in terms of time required for analysis, solvent noise and preparatory steps involved in the extraction of the drug from the formulation excipients for the estimation of drug contents. The retention times for valsartan and hydrochlorothiazide were 3.42 and 8.43 min, respectively. The typical chromatograms of the drugs are shown in fig. 1. The peak shapes of both drugs were symmetrical and asymmetry factor was less than 1.3.

**Fig. 1:**
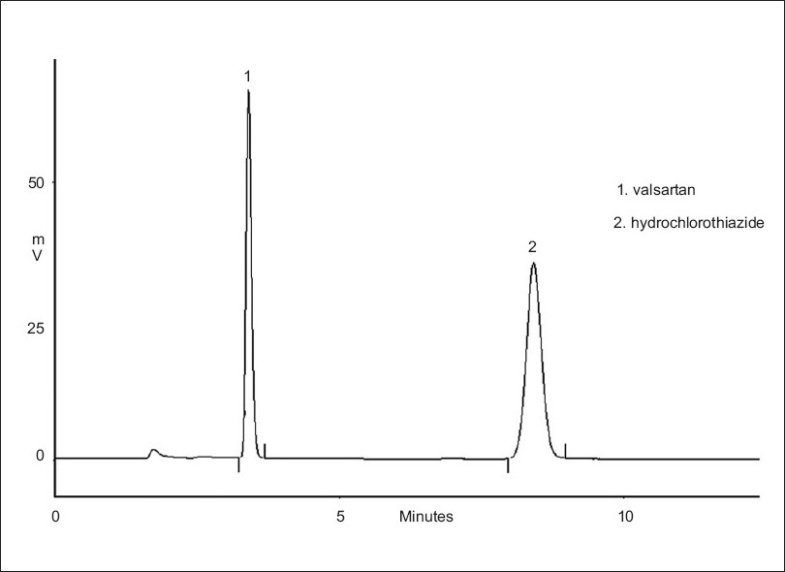
HPLC Chromatogram of valsartan and hydrochlorothiazide in tablets.

Pharmaceutical grade valsartan and hydrochlorothiazide were supplied by National Institute for the Control of Pharmaceutical and Biological Products and were used without further purification. All chemicals and reagents were of HPLC grade and were purchased from Jiangsu Hanbon Sci. & Tech. Co. Ltd.

HPLC system consisted of a pump (model LC-10AT plus). Manual injector was used. Loop used was of 20-μl capacity per injection. UV detector (model SPD-10AV plus) was used. Detection was carried out at 270 nm and the software used was Anstar Chromatographic Data System. Diamonsil (TM) C18 (200 × 4.6 mm, 5 μm) column was used. Different mobile phases were tested in order to find the best conditions for composition of mobile phase was determined to be methanol- acetonitrile- water- isopropylalcohol (22:18:68:2; v/v), and adjusted pH value to 8 with triethylamine. Flow rate was set to 1.0 ml/min.

Standard stock solution containing 0.01 and 0.00157 mg/ml of valsartan and hydrochlorothiazide, respectively was prepared by dissolving 10 and 1.57 mg of both drugs in 1000 ml of mobile phase. For estimation of valsartan and hydrochlorothiazide in tablets, an accurately weighed quantity of tablet powder equivalent to 10 mg valsartan and 1.57 mg hydrochlorothiazide were transferred to a 1000 ml volumetric flask, diluted with mobile phase, sonicated for 10 min and further diluted to 1000 ml with mobile phase. The solution was sonicated before it was used to analysis.

The plot of peak area of standard solutions versus concentration was found to be linear in the range of 5-150 μg /ml and 78-234 μg /ml for valsartan and hydrochlorothiazide respectively and coefficient of correlation (r^2^ ) was 0.9997 and 0.9998, respectively.

Amounts of drug powder equivalent to 100% of label claim, respectively of valsartan and hydrochlorothiazide were accurately weighed and assayed. The system repeatability was determined by six repeated applications. Repeatability of sample application and measurement of peak area were expressed in terms of relative standard deviation. Method repeatability was obtained from relative standard deviation by repeating six times during the same day for intraday precision. Intermediate precision was also done.

The reversed-phase HPLC method was developed to provide a specific procedure suitable for the rapid quality control analysis of valsartan and hydrochlorothiazide as referee method for the developed derivative method. To evalutate HPLC method robustness, a few parameters were deliberately varied; the parameters included pH of buffer and differents percentages of methanol and acetonitrile in mobile phase. Specificity of the methods was determined by the complete separation of valsartan and hydrochlorothiazide with other oarameters like retention time, asymmetry and capacity factor. The condition of the method was exactly effective and efficient.

To study accuracy, reproducibility and precision of the proposed method, recovery experiments were carried out. The average percentage recovery of valsartan and hydrochlorothiazide was 99.2% and 98.8% respectively. The sample recovery in the formulation was in good agreement with what was claimed on the label. System suitability parameters are given in Table 1. Assay in the tablet dosage form was found to be 98.6% of valsartan and 97.8% of hydrochlorothiazide. The method was simple and the run time given was 15 min. The proposed method gives a good resolution between valsartan and hydrochlorothiazide within a short analysis time (<15 min) and can be conveniently adopted for routine quality control analysis.

**TABLE 1:** SYSTEM SUITABILITY PARAMETERS

Parameters	Valsartan	Hydrochlorothiazide
Retention time (min)	3.42	8.43
Asymmetry	1.204	1.045
Theoretical plate	4238	5616
Resolution factor	-	9.0
Calibration range (μg /ml)	5-150	78-234
Correlation coefficient (r^2^)	0.9997	0.9998

To summarize, the method was evaluated in a mass of facets, such as best condition, linear relation including coefficient of correlation, robustness, accuracy, reproducibility and precision. All the parameters above showed that this method, which was fairly efficient and convenient, can be used to determine of Valsartan and Hydrochlorothiazide in tablets simultaneously.
